# Nutritional Value of the Larvae of the Black Soldier Fly (*Hermetia illucens*) and the House Fly (*Musca domestica*) as a Food Alternative for Farm Animals—A Systematic Review

**DOI:** 10.3390/insects15080619

**Published:** 2024-08-16

**Authors:** Welligton Conceição da-Silva, Éder Bruno Rebelo da Silva, Jamile Andréa Rodrigues da Silva, Lucieta Guerreiro Martorano, Tatiane Silva Belo, Carlos Eduardo Lima Sousa, Raimundo Nonato Colares Camargo-Júnior, Rubens Lima Andrade, Ana Gizela de Souza Santos, Katarina Cardoso de Carvalho, Adriny dos Santos Miranda Lobato, Thomaz Cyro Guimarães de Carvalho Rodrigues, Cláudio Vieira de Araújo, Jucelane Salvino de Lima, Kedson Alessandri Lobo Neves, Lilian Kátia Ximenes Silva, José de Brito Lourenço-Júnior

**Affiliations:** 1 Postgraduate Program in Animal Science (PPGCAN), Institute of Veterinary Medicine, Federal University of Para (UFPA), Castanhal 68746-360, PA, Brazil; eder.b.rebelo@gmail.com (É.B.R.d.S.); camargojunior@gmail.com (R.N.C.C.-J.); adrinysantos2@gmail.com (A.d.S.M.L.); thomazguimaraes@yahoo.com.br (T.C.G.d.C.R.); joselourencojr@yahoo.com.br (J.d.B.L.-J.); 2 Institute of Animal Health and Production, Federal Rural University of the Amazon (UFRA), Belem 66000-000, PA, Brazil; jamileandrea@yahoo.com.br; 3 Embrapa Eastern Amazon, Santarem 68010-180, PA, Brazil; lucieta.martorano@embrapa.br; 4 Department of Veterinary Medicine, University Center of the Amazon (UNAMA), Santarem 68010-200, PA, Brazil; tatianebelovet@gmail.com (T.S.B.); cadu34.medvet@gmail.com (C.E.L.S.); rubensandrade.medvet@gmail.com (R.L.A.); gizelamedvet@gmail.com (A.G.d.S.S.); katarinacc4@gmail.com (K.C.d.C.); 5 Department of Agricultural and Environmental Sciences, Federal University of Mato Grosso (UFMT), Sinop 78550-728, MT, Brazil; cvaufmt@gmail.com; 6 Institute of Biodiversity and Forests—IBEF, Federal University of Western Pará (UFOPA), Santarem 68040-255, PA, Brazil; jucelane.lima@ufopa.edu.br (J.S.d.L.); kedson_neves@hotmail.com (K.A.L.N.); 7 Institute of Veterinary Medicine, Federal University of Pará (UFPA), Castanhal 68740-000, PA, Brazil; silva_lilian@yahoo.com.br

**Keywords:** nutrition, sustainable alternative, insects, methane

## Abstract

**Simple Summary:**

The use of black soldier fly (*Hermetia illucens*) and house fly (*Musca domestica*) as an alternative for farm animal feeding has very important implications for sustainability since it does not require the use of resources such as water and land for production, in addition to contributing to food security, especially in locations with a reduced supply of animal feed. Thus, the use of these insects as a source of nutrients can reduce the need for supplementation and the use of grains and forages.

**Abstract:**

Dietary alternatives using insect-based products as an alternative for farm animal nutrition have been the object of study due to the high nutritional value of these feeds and the costs related to both their production and consequently their commercialization. Thus, the use of flies, especially larvae, has a high content of proteins and lipids (fat), as well as minerals and essential nutrients for development and growth, directly impacting the production of these animals, whether meat or milk. Therefore, the objective of this study was to compile data from the literature on the nutritional value of adults and larvae of Black soldier (*Hermetia illucens*) and housefly (*Musca domestica*) as a dietary alternative for animal feed. The Prisma checklist was used. After reviewing the data found in the literature, following the systematic review, it was noted that studies emerge that larvae of black soldier flies and domestic flies of the order Diptera obtain essential sources in the nutrition of ruminants, in addition to obtaining rapid digestibility, thus adhering to reproduction with high nutritional content, due to incident levels of protein, lipids, and minerals in *M. domestica* and *Lucilia sericata*, making it a target for inclusion in the diet of farm animals. In addition, it is concluded that both species are studied for their sustainable potential as well as for offering greater economic and nutritional viability when compared to ingredients present in production animal feed.

## 1. Introduction

The use of insect biomass has become an alternative for animal feed due to its high quality, low cost compared to feed, fast production, and low water and soil requirements, making it a sustainable option with lower greenhouse gas emissions [1,2]. 

Insects are able to convert waste from fruits and vegetables with low nutritional value into by-products with nutrient sources that are potentially marketable to industry, especially due to their high levels of protein and fat [3,4,5,6]. As it is a new product used for animal nutrition, this theme has become the target of studies in recent years given its numerous benefits both in relation to nutrition and for the economic sector [7,8,9,10].

Due to factors such as easy adaptation, large-scale production, and reduction in land use, this has become an option for ruminant nutrition due to its high sustainability when compared to protein products for soybean animal nutrition, since it requires chemical fertilizers and large tracts of land for cultivation [11,12,13]. 

Despite the numerous advantages mentioned for replacing vegetable proteins offered in animal feed, there is still little scientific research on the use of these products in the diet of ruminants [3,14]. Research on this topic is important because it serves as a subsidy to obtain information to assist in decisions regarding the regulation that authorizes the use of insects in the feeding of ruminants around the world [6,14,15].

The efficiency of the ruminant diet is determined by the amount of nutrients that reach the intestine and their bioavailability in terms of the appropriate values for animal production, as well as by the digestion process that occurs in the rumen by microorganisms available in the rumen microbiota [16,17,18]. 

Therefore, it is necessary to analyze the degradation of protein feeds to determine the potential of new foods in the nutrition of these animals, but the scarcity of data on the use of insects in ruminant nutrition as a protein option represents a negative point in terms of disrespecting the volume of information needed to validate the use in all parts of the world [19,20].

The nutritional value of larvae from the black soldier fly (BSF) and the house fly (*Musca domestica*) as a potential alternative food source for farm animals has gained significant attention. The BSF and house fly larvae (*Musca domestica*) have been widely studied and bred on a global scale due to their peculiarities as animal food sources. Sukmak et al. [21,22] highlighted in their preliminary studies the potential nutritional value of *Hermetia illucens* insects in terms of protein and lipids. Therefore, it is important to mention that the biological value and promise for use in animal feed need to be studied.

The BSF larvae are rich in protein content, typically ranging from 32% to 48% of dry matter [21]. According to Matsakidou et al. [23], evaluating the chemical composition of BSF, it was highlighted that the larvae obtained high levels of crude protein (CP) and 32% of dry matter compared to the pupae, which obtained 26.6% of dry matter. The BSF larvae contain a moderate amount of lipids (fat), usually around 12% to 42% of dry matter. This is due to the species and the development phase of the insect, which varies from larva to adult [20]. The fatty acid composition of these lipids is favorable, often rich in lauric acid and other medium-chain fatty acids [22]. The contents of minerals such as calcium, zinc, magnesium, phosphorus, and potassium range from 3.2% of mineral matter or ashes [23,24,25].

Both BSF larvae and house fly larvae offer promising nutritional profiles as alternative protein sources for farm animals [17,21,23,26]. BSF larvae are more extensively studied and widely recognized for their balanced nutrient composition and potential for use in animal nutrition [24]. However, further research is needed to fully evaluate the practicality and effectiveness of using house fly larvae as a sustainable feed alternative in animal farming as a generator of improved agricultural practices [10,26].

Fly larvae also have chitosan, which is easily digested in an acidic environment and can be found in the deacetylated form of chitin present in nature, mainly in the exoskeleton of insects, which has amino acids and antimicrobial and antioxidant properties, unlike chitin, which is more unstable and has low digestibility, which can make it a toxic component in animal feed [19,22,26].

In addition, the methods used to evaluate other protein alternatives for farm animals, such as in vitro cultures, are reasons for questioning, given the possible overestimation of the data obtained by the loss of particles during the sample processing process and not by their degradation [16,27]. 

In view of this, several studies involving the use of insects in the total or partial feeding of numerous species have been developed in several countries, but in the case of ruminants, this interest is still limited due to the risk of developing some diseases in the herd, such as Bovine Spongiform Encephalopathy, known as mad cow, thus prohibiting the use of animal protein for supplementation of production animals by the European Union [5,28,29,30,31,32,33,34,35,36,37].

Thus, understanding the nutritional value of different species of flies helps producers and researchers make decisions in the face of food scarcity in different parts of the globe, enhancing a sustainable and economically viable diet. Added to this, seeking the study of art on this theme facilitates the understanding of the gaps filled and those missing within the global scenario. Based on this information, the objective of this study was to compile data from the literature on the nutritional value of black soldier (*H. illucens*) and housefly (*M. domestica*) larvae as a dietary alternative for animal feed.

## 2. Material and Methods

This review was developed through a bibliographic survey in the Web of Science and Scopus databases, which are considered solid scientific databases with a high storage capacity for high-quality articles. In this study, we used the following search keywords: “Legislation on the use of fly larvae in animal diets”, “BSF meal in animal diets”, “Fly larvae in animal diets”, and “Nutritional value of flies in animal diets”. 

The inclusion criteria were full text, written in English, Portuguese, and Spanish, and that presented a discussion regarding the nutritional value of flies in the diet of ruminants. Articles were excluded when they did not fit the objective of the study. 

In this study, we chose not to use time or geographic periods during the searches. Specify the methods used to decide whether a study met the review’s inclusion criteria, including the number of reviewers who analyzed each record and report retrieved, whether they worked independently, and, if applicable, details of the automation tools used in the process. 

The studies were separated according to title and abstract, after which they were analyzed in triplicate by the authors independently, avoiding interference. The variables retrieved and analyzed from the manuscript were the year of publication of the study, title, species, breed, number of animals in the study, experimental design, and diet. 

**Figure 1:**
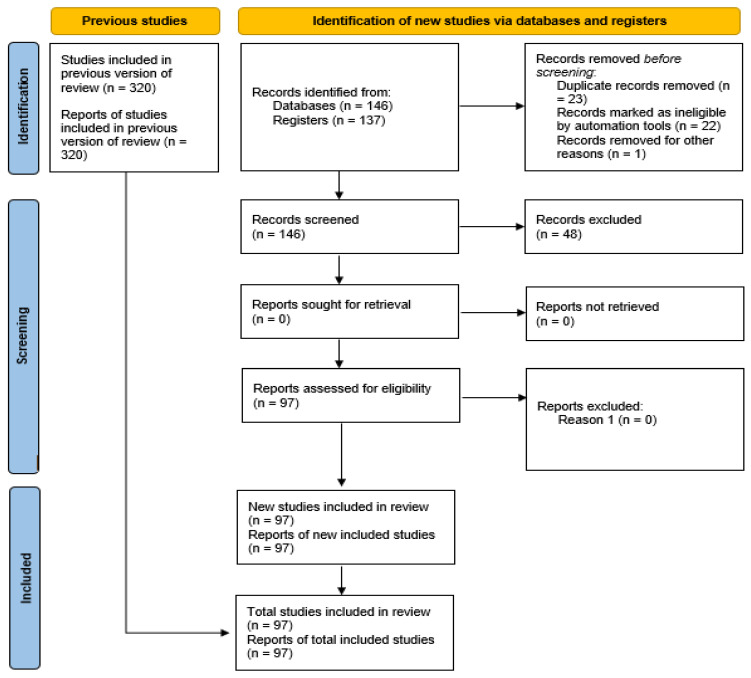
Flowchart used to select the articles used in the systematic review, as established in the prism [39].

**Figure 2:**
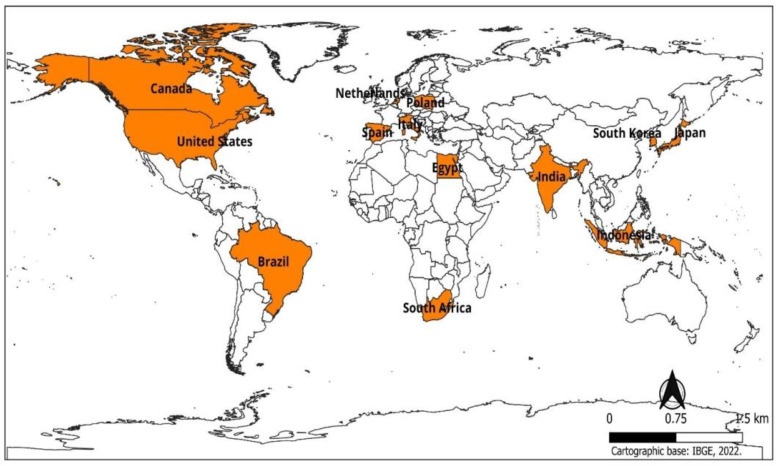
Map of countries that have researched the use of flies in livestock feed.

Thus, we considered the entire literature review until the end of the database searches. A total of 97 references were cited in this review. In this study, the data were organized in spreadsheets and subsequently evaluated, all presented in table formats and graphs to facilitate the reader’s understanding. The same methodology was used in the studies by Camargo-Júnior et al. [38]. The Prisma [39] checklist (Table S1) was used (Figure 1); note the Prisma Flowchart presenting the selected criteria and articles throughout the review. Figure 2 shows the countries that researched the use of flies in livestock feed. 

Table 1 shows the different experimental studies that have been carried out using different species of farm animals, adding the year, authors, title of the article, species, breed, number of animals (NA), and the experimental design and diet.

## 3. Literature Review

### 3.1. Regulations on the Use of Insects in Ruminant Feeding

The legislation regarding the use of processed animal proteins (PAPs) is diverse around the world. While developed countries prohibit their use in ruminant nutrition, underdeveloped countries do not have specific legislation on the use of these products [59]. 

This prohibition is an obstacle to the development of research on by-products derived from insects in the feed of these animals, given the risk of developing mad cow disease, but in American countries it is common to find fish meal, bone meal, and meat produced from by-products of other animals in ruminant feed [7,29,58,60].

Despite these conflicts in relation to legislation and the different locations of the globe for the use of feed based on insect meal, the interest of researchers is increasingly notorious in this productive sector, as well as that of feed mills, due to the growing demand for feed for ruminants to meet the need in production for the consumption of food products for these animals [61,62,63]. It is estimated that the use of these products in the feed of monogastric animals will occur soon, and with this, there will be significant changes in the legislation favoring the use of insects and their by-products in the feed of ruminants in the world [64,65,66,67].

### 3.2. Fly Larvae

#### 3.2.1. Chemical Constituents

The chemical constitution of the BSF depends on its diet, it has many nutrients and is rich in proteins and fats. For example, larvae fed on poultry manure have 40 to 44% CP and fats with 15 to 25% [68]. The amount of minerals is high, but the content of dry matter, calcium, and phosphorus varies according to the species [40,68,69]. The larvae are used in animal feed, cut in order to remove intracellular fat in order to have a low-fat meal [70]. It is possible to see a higher value of CP and crude fat in these larvae, making them a potential for animal production [71,72,73,74,75,76].

##### Housefly Larvae Meal and Housefly Pupae Meal

In the 1960s, the use of the housefly (*M. domestica* Linnaeus, 1758) as a food source for farm animals was observed and studied [77,78]. The fact that these flies have a varied diet of degrading waste makes them a very nutritious source. This species is the most common of all and has great importance in the transmission of diseases due to its way of feeding. In reproduction, they need an appropriate environment with high temperatures (>25 °C). 

Female flies lay 500 to 600 eggs depending on their environmental conditions and look for more humid places to lay their eggs. They hatch in about 8 to 12 h, the larval stage lasts 5 days, and the pupal stage lasts 4 to 5 days. In the adult stage, they consume mainly degrading waste this feeding is done with regurgitation of saliva, so they end up transmitting some microorganisms, and when mating occurs, the flies lay eggs at the time of feeding. The larvae do not necessarily need large amounts of food to develop; for example, waste with 450 g of fresh manure feeds about 1500 larvae [64]. 

In the rearing of larvae, it is necessary to have a suitable environment with an attractive diet and temperatures of 25 to 30 °C and a humidity of up to 60 to 75% ideal for the growth of the larvae [78]. The researchers used various types of feed, with poultry manure being the most used [64,79], and others already mentioned, such as cattle intestine remains [80], fish intestine [81], and pig manure [82,83]. This residue is deposited in a suitable container and sprayed with water to send it to an appropriate, humid environment.

##### Quantitative and Qualitative Aspects of Animal Performance

Studies are still needed to present more accurate results on the use of insects in the diet of animals. Research on how to replace feed and include, for example, cricket meal in the diet of post-weaning goats has not yet yielded a complete result [84]. 

The use of oils obtained from *H. illucens* as a substitute for soybean oil can benefit energy production in ruminants [56], increasing health-promoting acids in the animal. The use of insect fat in ruminant feed is variable, as the composition of fatty acids is different depending on the species analyzed, its stage of development, and the diet on which the insect is being fed [85,86]. 

The amount of fatty acids present in insects is interesting in ruminant feed, as it has an impact on the quality of derived products. Analysis of the chemical components and nutritional benefits of different insects as animal feed.

#### 3.2.2. Chemical Composition and Nutritional Value of *H. illucens* Larvae

**Figure 3:**
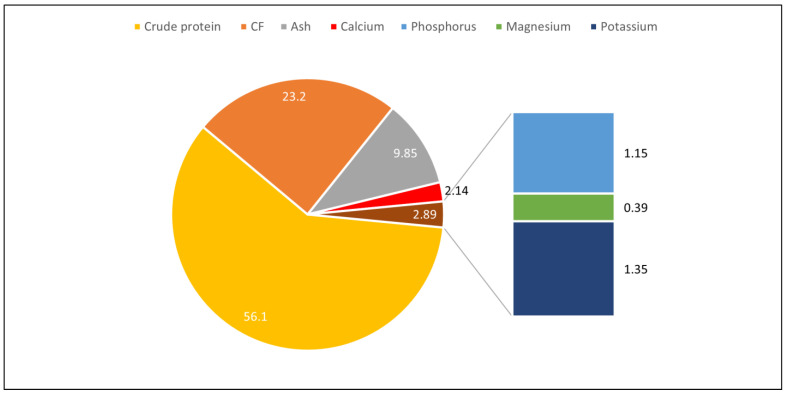
Nutritional composition of BSF larvae. Adapted from Marco et al. [44]; Jayanegara et al. [87]. CF = crude fiber.

In the breeding of black soldier flies, various residues from both animals and vegetables, as well as coffee pulp, are used in their feed [47]. In the adult stage, they are black in color and reach up to 20 mm in length. According to the nutritional composition of the larvae, they are rich in protein, calcium, phosphorus, magnesium, potassium, zinc, manganese, and iron. These nutrients vary according to the stage; if it is larval or adult, the values are different, but always in significant amounts. Several studies have demonstrated the types of nutrients and proteins in diets performed by different researchers with BSF larvae. The nutritional composition of BSF larvae can be seen in Figure 3. 

BSF larvae. The substance of matter in BSF is much higher (34.9% to 44.9%), which modifies the BSF larvae so that they develop easily and are available at a cheaper cost-benefit compared to other good products intended for feeding farm animals. Approximately, the BSF larvae integrate about 41.1% to 43.6% crude protein (CP), 15.0% to 34.8% Ethereal extract (EE), 7.0% to 10% crude fiber (CF), ash 14.6% to 28.4%, and 5278.49 kcal/kg Crude energy (CE) [44,87]. 

BSF larvae are eminent in Ca (5% to 8%) and P (0.6% to 1.5%). In addition, the mineral profile includes (0.6%), Fe (0.14% to 14%), Mn (24.6%), Mg (0.39%), sodium (Na, 0.13%), K (0.69%), and Zn (11%) [40,47]. According to Cullere et al. [47], indispensable essential amino acids (EAA) such as leucine (Leu) and valine (Val) are abundant in BSF larvae (BSF). 

Marco et al. [44] showed a lower amount of arginine (Arg), histidine (Hist), lysine (Lys), and methionine (Meth) in diets based on BSF larvae when compared to previous research; on the other hand, the amino acids isoleucine (Isoleu), phenylalanine (Phy), and threonine (Thar) were found to be higher.

##### *H. illucens* Larvae and Their Impacts on Ruminant Digestibility 

Probable types of protein source insects for feeding ruminants such as cattle include Jamaican field crickets, BSF larvae, and mealworms. Jayanegara et al. [87] employed these three types of insects for the nutrition of Frisian Holstein cattle. They describe that the insect feeds regularly 40% more dry matter (DM) than the Jamaican field cricket; in addition, the insect feed was remarkable in superiority to fiber content to the soybean meal (SBM). 

In the supply of food for insects, black soldiers found remarkably higher neutral detergent fiber (NDF) and acid detergent fiber (ADF) indexes in the larvae with regard to in vitro ruminal fermentation, the facial appearance and digestibility of the insect, and SBMs demonstrate the agglomeration of similar total volatile FA, the pretext of insoluble CP in detergent, unstable acid in the in vitro digestibility of dry matter, and the digestion of organic matter molybdenum in insect feed than in SBM. 

The food produced by insects produces less gas because of the higher fragments of fiber and ether extract (EE) than SBM. In this way, the in vitro system of the gas is designed, particularly through the fermentation of carbohydrates, and the binding of EE in producing the gas is negligible. The high level of NH_3_ in the Jamaican field stands out with the highest CP content. The exclusion of proteolysis and the breakdown of complex molecules into simpler products in the organism resulted in physiological degradation by proteolytic microorganisms rich in enzymes in the ruminal form of NH_3_ [88]. 

The composition of NH_3_, an alkaline molecule formed from the nitrogen cycle by the action of the decomposition of organic matter by microorganisms in the digestive system, more precisely in the rumen, is not totally dependent on the concentration of CP; therefore, it depends on the rate of enzymatic hydrolysis, rate of passage, formation of microbial proteins, and renal absorption of ammonia, through blood flow and the rumen wall [89,90,91,92].

The analysis of the ruminant animals put the ration containing all the insects and visualized that the cattle eliminated a minimum fraction of methane than those fed on the basis of SBM, and the lower digestion of the insect succeeded in minimal production of hydrogen, which is an essential substrate for the production of methanogenesis, the final step in the global process of anaerobic degradation of organic matter biodegradable into methane [87,93,94].

The importance of the BSF larvae in cattle is that they have high nutritional value and help in the diet of the animals. Their high palatability and digestibility make it possible to accelerate products through the agricultural industry with a low cost-benefit ratio in biomass and a higher quality in protein [95]. 

**Figure 4:**
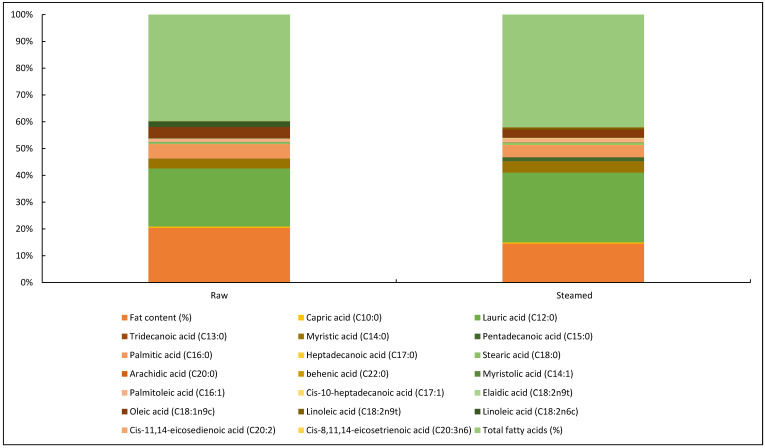
Fatty acid content of 15-day-old BSF larvae kept in organic waste.

**Figure 5:**
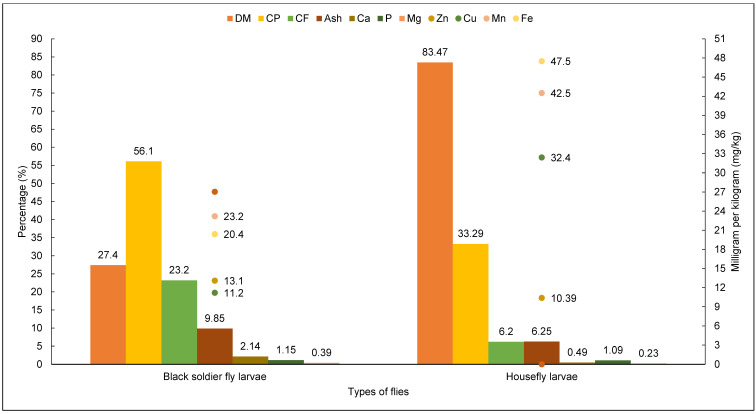
Chemical composition and nutritional content of BSF larvae and housefly larvae. DM, dry matter; CP, crude protein CP; CF, crude fiber; Ash; Ca, calcium; P, phosphorus; Mg, magnesium; K, potassium; Na, sodium; Zn, zinc; Cu, copper; Mn, manganese; Fe, iron. Adapted from St-Hilaire et al. [40], Moreki et al. [90], Rumpold et al. [91], and Campbell et al. [92]. The chemical composition of BSF larva meal can be shown in Figure 6. It is possible to observe a higher percentage of dry matter (94.69%) and CP (55.83%), and by minerals, a higher percentage of iron (71.50%).

Figure 4 shows the fatty acid content of 15-day-old BSF larvae kept on organic waste, showing the percentage of fatty acids in the raw and steamed food. Figure 5 shows the nutritional values for dry matter (DM), crude protein (CP), crude fiber (CF), ash, calcium (Ca), phosphorus (P), magnesium (Mg), potassium (K), sodium (Na), zinc (Zn), copper (Cu), manganese (Mn), iron (Fe), and the chemical composition of BSF larvae (*H. illucens*) and housefly larvae (*M. domestica*).

**Figure 6:**
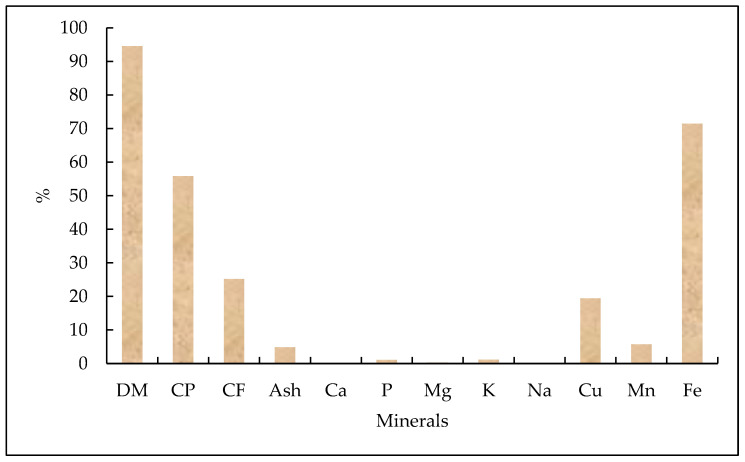
Nutritional information on fly larvae flour. DM, dry matter; CP, crude protein; CF, crude fiber; Ash; Ca, calcium; P, phosphorus; Mg, magnesium; K, potassium; Na, sodium; Cu, copper; Mn, manganese; Fe, iron. Adaptado de Marono et al. [45] e Song et al. [93].

According to [96], the results of the research showed that the in vitro digestibility of organic matter (OM) and HLF fly larvae was 83.2% and 84.3%, respectively. In addition, the centesimal analysis revealed the following values (Figure 6).

#### 3.2.3. Chemical Composition and Nutritional Quality of *M. domestic* Larvae

Housefly larvae are rich in energy, so protein and micronutrients are part of this compound, in addition to iron and zinc, which contribute to better effectiveness. In general, HFL meals have high amounts of Lys, Thr, and Met and can be added to low-protein cereals and pulse-based diets for ruminants [42,92,94,95].

According to Hwangbo et al. [41], the HFL fly is rich in CP (63.99% of DM) and ether extract (24.31% of DM). Due to drying techniques and the age of the larvae, the CP and ether extracts may differ; the CP content decreases and the EE content increases with age. According to Makkar et al. [3] and Khan et al. [95], the nutritional evaluation of HFL was evaluated and demonstrated that crude protein is the largest component with 76.23%, followed by ether extract with 14.39% and ash with 7.73% (degree of fattening: 4877.23 kcal/kg).

Research has observed that HF pupae and larvae have apparent metabolic energies of 3398.77 kcal/kg and 3618.51 kcal/kg, respectively. The larvae showed high levels of Ca, P, and metabolic energy (4140 kcal/kg) compared to MCS (2250 kcal/kg) [96]. 

Linoleic acid was identified in concentrations between 26.3% and 36.3% in relation to the total fat composition in diets containing the addition of pupae and larvae of *H. illucens*, respectively. Comparatively, the larvae have a significant content of palmitoleic acid in direct comparison with the pupae; however, for this species, the high presence of essential fatty acids, such as oleic acid and linoleic acid, is remarkable. Compared to soybean meal (SBM), larval analyses signal higher levels of amino acids (AAs) and essential amino acids (EAAs). The high levels of EAAs, including leucine, phenylalanine, tryptophan, and valine, of the larvae are noteworthy, but they show less significant amounts of methionine and serine in relation to SBM [97]. 

In the diet of ruminants, the use of larvae and pupae can be an ally, mainly because the pupae of *H. illucens* (BSF) showed high levels of CP (CP), phosphorus (Phy), lysine (Lys), and methionine (Meth) compared to the meal larvae (*Tenebrio molitor*). However, when compared in terms of nutritional value, the meals had similar characteristics to bone meal and meat meal, as well as fish meal, and were superior to whole soybean meal (SBM).

The aforementioned study highlights that larvae have the highest CP content in raw egg concentrates, reaching 45.8%, and the highest EE content, with 19.3%. The larvae that feed on the chopped mangoes have a higher amount of ash (7.1%). The maximum CP value was observed 48 h after harvest (58.0%) (Table 2). Most were EE and CP 120 h after harvest (24.6 and 7.6, respectively) in the observatory analysis [97].

### 3.3. Effect of Housefly Larvae on Nutrient Digestibility in Farm Animals

**Figure 7:**
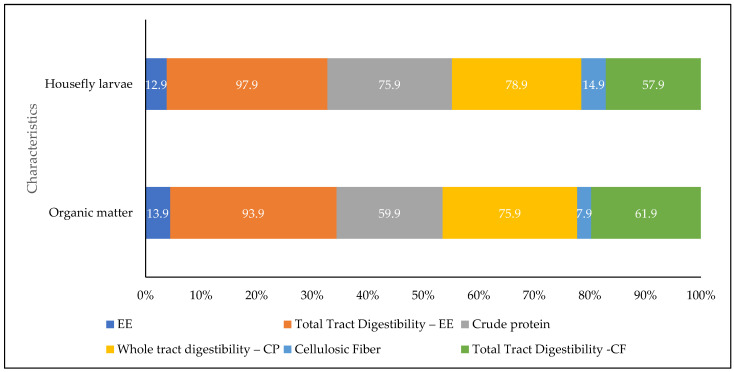
Centesimal analysis of housefly larvae. Note: Measured in kcal/kg. CF = crude fiber; EE = Ethereal extract; CP = crude protein; HFL = housefly larva. Adapted from Ukanwoko et al. [97].

The nutritional analysis of the feeding of housefly larvae showed comparable values to most feed ingredients with a high protein content. The larval diet consisted of 60% protein, a significant amount essential for the growth and development of the larvae, in addition, it had a balanced profile of AA and 20% fat, with 57% monounsaturated FA and 39% saturated FA. However, the larvae were deficient in omega-3 FA [96,97] (Figure 7). 

Throughout this review, we have noted that the use of insects to feed farm animals is proving to be an alternative to foods with many benefits, both in terms of sustainability and animal nutrition and food. Studies show that the use of insects and their larvae is potentially nutritious due to the bioavailability of nutrients present in their composition, such as proteins, fatty acids, vitamins, and minerals.

Thus, in this study, there are various nutritional benefits such as the development rate, improvement of intestinal health, and food efficiency. Thus, despite the need to evaluate the quality of the production processes of these foods and the concern with possible toxins present in substrates, the results are promising and highlight the need for research associated with the optimization and standardization of the production and management systems of these insects to ensure food safety and animal health.

## 4. Limitations and Future Perspectives 

Because the nutritional value of flies is high, there are several restrictions to be pointed out. Firstly, the availability and quality of flies as food sustenance can vary considerably according to factors such as the production environment and the food set used. In addition, the action of pathogens and environmental contaminants present in flies can pose a threat to the health of ruminants, requiring rigorous processing techniques and quality inspection.

The use of biotechnology with methods that make it possible to improve the use of insects in animal feed in order to enable the bioavailability of essential nutrients such as proteins, vitamins, minerals, and fatty acids to be better utilized. Furthermore, the production costs of these products are lower compared to traditional methods of producing animal food, as the substrates intended for feeding these insects can come from agricultural and food waste, contributing in a sustainable way, reducing unwanted environmental impacts, and ensuring food security.

Furthermore, the use of supplements that aim to improve the quality of food from insects can benefit animal health and productivity. The use of probiotics can promote intestinal health by favoring the digestion and nutrient absorption processes of farm animals; thus, the use of these techniques makes it possible to add unique value to the industry of food products from insects intended for animal nutrition.

Some limitations related to the use of insects in animal feed are due, in particular, to cultural prejudices associated with the production of these foods and their supply. In some countries, the main obstacles to insect-based animal nutrition are related to problems related to regulations associated with legislation aimed at animal nutrition, as well as barriers related to policy and education campaigns on the adoption of these practices.

Furthermore, the use of different substrates intended for feeding insects can impact the nutritional composition of the final products, making it difficult to standardize these foods, so it is still necessary that better technologies and infrastructures be adopted so that the production of insects for animal feeding on farms is improved, such as the adequate management of resources and by-products intended for feeding insects, in order to try to achieve the improvement and standardization of insect feeding, yet the adoption of these practices must be carried out appropriately so that negative environmental impacts are avoided. Thus, the development of continuous research and technologies can be considered to minimize and overcome limitations, making the use of insects in feeding farm animals a viable alternative both economically and sustainably.

Future insights in this sector include the growth of effective large-scale production pathways for flies, aiming to improve their nutritional value and decrease health hazards. Continuous studies on the nutritional composition of the various species of flies linked to the impact on the health and efficiency of ruminants are essential to fully investigating their capacity as a dietary source. Therefore, the search for revolutionary processing technologies, such as drying and disinfection, contributes to the development of the safety and quality of flies as a food source for ruminants.

## 5. Conclusions

Studies emerge that larvae of black soldier flies and domestic flies of the order Diptera obtain essential sources in the nutrition of ruminants, in addition to obtaining rapid digestibility, thus adhering to reproduction with high nutritional content due to the incident levels of protein, lipids, and minerals of *M. domestica and Lucilia sericata*, making it a target for inclusion in the diet of farm animals. In addition, it is concluded that both species are studied for their sustainable potential as well as for offering greater economic and nutritional viability when compared to ingredients present in animal feed production. In addition, the methane reduction was evident in a study using BSF and housefly larvae, minimizing carbon levels in animal production.

## Figures and Tables

**Table 1:** Information about the articles used in the review.

^¥^ Year	Authors	Title	Species	Breed	NA	Experimental Design and Diet
2007	St-Hilaire et al. [40]	Fly Prepupae as a Feedstuff for Rainbow Trout, *Oncorhynchus mykiss*.	Fish—Rainbow trout	*Oncorhynchus mykiss*	¥¥	Three test diets were formulated, which consisted of partially replacing the fishmeal of the control diet with larvae of different flies: *Hermetia illucens* prepupae (25% and 50% replacement) and *Musca domestica* pupae (25% replacement).
2009	Hwangbo et al. [41]	Utilization of house fly maggots, a feed supplement in the production of broiler chickens.	Male Cutting Chick	Ross Lineage	150	A total of 600 birds were divided into five groups, one fed a control diet and the other with a basal diet supplemented with 5.0, 10.0, 15.0, and 20.0% housefly larvae; however, on day 35, 30 birds from each treatment group were chosen for the analyses.
2013	Pieterse and Pretorius [42]	Nutritional evaluation of dried larvae and pupae meal of the housefly (*Musca domestica*) using chemical and broiler-based biological assays.	Chick	Cobb 500 lineage	120	The animals were distributed in metabolic cages, with five chicks per cage, totaling 24 cages used in the experiment, in which, at 25 days of age, they were fed one of three diets, which were based on corn (the reference diet), larvae, or pupae.
2014	Schiavone et al. [43]	Nutrient digestibility of *Hermetia illucens* and *Tenebrio molitor* meal in broiler chickens.	Chickens	Ross Lineage 706	30	The diets were randomly allocated to cages on the 26th day, consisting of a control diet and two experimental diets with larvae, in which 25% (*w/w*) of the basal diet was replaced by *Tenebrio molitor* larvae meal or *Hermetia illucens* larvae meal.
2015	Marco et al. [44]	Nutritional value of two insect larval meals (*Tenebrio molitor* and *Hermetia illucens*) for broiler chickens: Apparent nutrient digestibility, apparent ileal amino acid digestibility, and apparent metabolizable energy.	Poultry (Chick)	Ross 708 Lineage	90	On day 19 of life, the birds were divided into three groups: the Control Group; Group TM (*Tenebrio molitor*); and Group HI (*Hermetia illucens*) to evaluate digestibility coefficients.
2015	Marono et al. [45]	In vitro CP digestibility of *Tenebrio molitor* and *Hermetia illucens* insect meals and its correlation with chemical composition traits.	In vitro	¥¥	¥¥	Twelve samples of insect meal (*Tenebrio molitor* and *Hermetia illucens*) obtained from small producers and submitted to centesimal and CP analysis by the two-step enzymatic method were analyzed in vitro.
2016	Bovera et al. [46]	Use of *Tenebrio molitor* larvae meal as a protein source in broiler diet: Effect on growth performance, nutrient digestibility, and carcass and meat traits.	Chicken	Shaver	32	They were divided into 8 replicates of 5 birds, totaling 40 birds in each group (2), of which, after 62 days of age, 16 were slaughtered per group. The first group received a diet based on corn and soybean meal, and the other group received meal from *Tenebrio molitor larvae*.
2016	Cullere et al. [47]	BSF as a dietary protein source for broiler quails: apparent digestibility, excreta microbial load, feed choice, performance, carcass, and meat traits.	Quails	*Coturnix coturnix japonica*	15	450 birds were divided into groups that received a control diet (C) and two diets (H1 and H2) corresponding to inclusion levels of 10% and 15% of meal from defatted larvae of the BSF, of which 15 were randomly chosen for the analysis of the groups.
2016	Jin et al. [48]	Supplementation of dried mealworm (*Tenebrio molitor larva*) affects growth performance, nutrient digestibility, and blood profiles in weaning pigs.	Piglets	Mestizo	120	They were divided into five feeding groups, in which there was a control group based on corn-barley-soybean meal and four groups with a diet of 1.5%, 3.0%, 4.5%, or 6.0% dry meal powder for 35 days after weaning.
2017	Borrelli et al. [28]	Insect-based diets, a promising nutritional source, modulate gut microbiota composition and SCFA production in laying hens	Pruning chickens	Lohmann Brown Classic	24	They were divided into two groups with different diets, one based on corn and soybeans and another with a defatted flour of *H. larvae* of *illucens*.
2017	Valle et al. [49]	Dietary chitosan improves nitrogen use and feed conversion in diets for mid-lactation dairy cows.	Cows	Holstein Breed	24	Cows were segregated into six groups based on the presence of a rumen cannula, days in lactation, and milk production. These animals were randomly allocated to a treatment sequence: control; chitosan; soybean oil, and chitosan + soybean oil.
2017	Dias et al. [50]	Increasing doses of chitosan to grazing beef steers: Nutrient intake and digestibility, ruminal fermentation, and nitrogen utilization.	Steers	Rumen cannulated crossbred steers	5	Five different treatments were designated, in which each bovine was randomly raised. For 21 days, the animals received doses of chitosan added to the concentrate fed to the animals, with doses of 0, 400, 800, 1200 or 1600 mg/kg of dry matter (DM) of the concentrate
2017	Jayanegara et al. [11]	Use of BSF larvae (*Hermetia illucens*) to substitute soybean meal in ruminant diets: An in vitro rumen fermentation study.	In vitro	Rumen microorganisms from a non-lactating fistulated Friesian-Holstein cow	¥¥	Evaluation of rumen fermentation in vitro, in which soybean meal was replaced by BSF larvae in a Napier grass diet, with analysis of six treatments with different percentages of dietary treatments.
2017	Jayanegara et al. [12]	Evaluation of some insects as potential feed ingredients for ruminants: chemical composition, in vitro rumen fermentation, and methane emissions.	In vitro	Rumen fluid collected from a Friesian Holstein cow with rumen fistula	¥¥	In vitro ruminal fermentation tests were carried out, in which Jamaican cricket, mealworm, and larvae of the BSF at 1 and 2 weeks of age were used to evaluate methane emission, chemical composition, in vitro ruminal fermentation, and digestibility.
2017	Marono et al. [51]	Productive performance and blood profiles of laying hens fed *Hermetia illucens* larvae meal as a total replacement of soybean meal from 24 to 45 weeks of age	Laying hens	Lohmann Brown Classic	108	The chickens were equally segregated into two groups, with distinction in feeding, the first based on soybean meal and the other on defatted meal from *Hermetia illucens larvae*.
2018	Rashmi et al. [13]	Effect of dietary incorporation of silkworm pupae meal on in vitro rumen fermentation and digestibility	In vitro—rumen content of three steers	Mestizos	¥¥	From the analysis of eleven mixtures, and before feeding and morning water supply of the three steers, the inclusion of defatted silkworm pupae meal (DSWP) in rumen fermentation and in vitro digestibility were analyzed.
2019	Benzertiha et al. [29]	*Tenebrio molitor* and *Zophobas morio* full-fat meals in broiler chicken diets: Effects on nutrient digestibility, digestive enzyme activities, and cecal microbiome.	Broiler chicks (Female)	Ross Lineage	600	Six experimental groups were used in the present study with two different levels of yellow flour and whole wheat superflour (0.2% and 0.3%), a positive control with the addition of salinomycin, and a negative control.
2019	Biasato et al. [30]	Partially defatted BSF larval meal inclusion in piglet diets: effects on growth performance, nutrient digestibility, blood profile, gut morphology, and histological features	Piglets	Topigs	48	Three different feeding treatments, with four boxes as replicates for each treatment and four animals per box. FSB larval meal was added at increasing levels (0%, 5%, and 10%) in diets formulated for two feeding phases: phase I (from day 1 to day 23) and phase II (from day 24 to day 61).
2019	Cullere et al. [31]	*Hermetia illucens* larvae reared on different substrates in broiler quail diets: effect on the physicochemical and sensory quality of the quail meat.	Quails	¥¥	300	They were divided into three experimental groups: control; soldier fly larvae reared on conventional substrate; and soldier fly larvae reared on substrate composed of 50% chicken layer must and 50% fish offal.
2019	Fontes et al. [32]	Digestibility of insect meals for Nile tilapia fingerlings.	Male Nile tilapia fingerlings	Oreochromis niloticus	900	Six dietary treatments were used (control—no insect meal and five insect flours) in three replications (cylindro-conical fiberglass tanks with a capacity of 250 L), each containing 50 fingerlings.
2019	Gasco et al. [5]	Effect of dietary supplementation with insect fats on growth performance, digestive efficiency, and health of rabbits.	Rabbits	Mestizo	200	A total of 12 animals from the following groups were randomly evaluated: a control diet with 1.5% soybean oil; and four experimental diets, partially (50%) or totally (100%) replaced by fats, soldier fly larvae, and yellow cascudinha larvae.
2019	Yoo et al. [52]	Nutrient ileal digestibility evaluation of dried mealworm (*Tenebrio molitor*) larvae compared to three animal protein by-products in growing pigs.	Pigs	Mestizo	12	The pigs were surgically fitted with simple T cannulas. After the recovery period, the animals were allocated to four different treatments, which consisted of different diets, one of which was based on *Tenebrio molitor*.
2020	Chemello et al. [34]	Partially defatted *Tenebrio molitor* larva meal in diets for grow-out rainbow trout, *Oncorhynchus mykiss* (Walbaum): Effects on growth performance, diet digestibility, and metabolic responses	Fish—rainbow trout	Oncorhynchus mykiss Walbaum	252	The animals were randomly divided into twelve tanks and fed four experimental diets containing different levels of *Tenebrio molitor* larval meal inclusion (0%—control, 25%, 50%, and 100%—positive fishmeal replacement test).
2020	Dabbou et al. [35]	Yellow mealworm (*Tenebrio molitor L.*) larvae inclusion in diets for free-range chickens: Effects on meat quality and fatty acid profile.	Free-range chickens	Hubbard Label Hybrid Chickens	140	Feeding trial with two dietary treatments, a control group and a group with *Tenebrio molitor larval meal*, in which at 97 days of age ten, from each group were slaughtered for evaluation.
2020	Jayanegara et al. [53]	Fatty acid profiles of some insect oils and their effects on in vitro bovine rumen fermentation and methanogenesis.	In vitro	Rumen microorganisms—fistulated Ongole crossbred cattle	¥¥	Experimental trial of in vitro ruminal fermentation, with the objective of evaluating the effects of oils from different insect species on rumen fermentation and methane production
2021	Ahmed et al. [20]	Insects as novel ruminant feed and a potential mitigation strategy for methane emissions	Cows	Holstein cows	2	Six experimental groups with different combinations of Klein grass hay, soybean meal, and edible insects were used for the rumen fluid study (rumen fistulated cows)
2021	Matin et al. [54]	True metabolizable energy and amino acid digestibility in BSF larvae meals, cricket meal, and mealworms using a precision-fed rooster assay.	Gauls	Single Comb White Leghorn	¥¥	In six experiments, seven different insect meals were analyzed to determine their chemical and nutritional composition.
2021	Kar. et al. [55]	Local intestinal microbiota response and systemic effects of feeding BSF larvae to replace soybean meal in growing pigs.	Pigs	Wild boar	16	The animals were distributed equally and randomly into two groups, in which they were fed for three weeks with isocaloric and isoprotein experimental diets prepared with soybean meal or *Hermetia illucens* meal.
2022	Hervás et al. [56]	Insect oils and chitosan in sheep feeding: Effects on in vitro ruminal biohydrogenation and fermentation.	In vitro—microrganisms ruminais de ovelhas	¥¥	¥¥	Ten treatments were used, divided between one control without the addition of oil plus four oil supplements × absence or presence of chitosan.
2022	Toral et al. [14]	Insects as an alternative feed for ruminants: comparison of protein evaluation methods.	Intro—Rumen content of four sheep	Merino	¥¥	In vitro analysis of the potential of four insect species (*Tenebrio molitor, Zophobas morio, Alphitobius diaperinus, and Acheta domesticus)* as alternative protein sources for ruminants.
2023	Tansil et al. [57]	Evaluation of standardized ileal digestibility of amino acids and metabolic availability of methionine, using the indicator amino acid oxidation method, in BSF larvae (*Hermetia illucens*) meal fed to growing pigs.	Pigs	Yorkshire	Experiment 1: 6 Experiment 2:7	Two experiments were carried out with cannulated male pigs in order to evaluate the ileal digestibility and metabolic availability of methionine in partially defatted BSF larvae (PD-BSF) meal.
2024	AbdelHakeam, et al. [58]	Effect of insect meal as a substitute for soybean meal on performance of Ossimi lambs.	Lamb	Ossimi	40	Partial replacement in the diet of 0, 10, 20, and 30% of soybean meal with an equal portion of Oriental Wasp Flour.

Note: NA = number of animals. ¥ = year of publication of the manuscript. ¥¥ = information not provided in the literature.

**Table 2:** Percentage of crude protein and ether extract in mango and raw eggs.

Nutrients	Raw Eggs		Crushed Mango	
	Immediate	120 h	Immediate	120 h
Crude protein	45.8%	7.6%	39.5%	6.1%
Ethereal extract	19.3%	24.6%	19.1%	5.8%

## Data Availability

The original contributions presented in the study are included in the article; further inquiries can be directed to the corresponding author.

## Supplementary Materials

The following supporting information can be downloaded at: https://www.mdpi.com/article/10.3390/insects15080619/s1, Table S1: PRISMA checklist. Reference [39] is cited in the Supplementary Materials.

## Nomenclature

**Abbreviation**	**Definition**
CP	Crude Protein
PAPs	Processed animal protein
EE	Ethereal extract
SBM	Soybean meal
VFA’s	Volatile Fatty Acids
pH	Hydrogen potential
mg	Milligram
mm	Millimeters
BSFL	BSF larvae
CF	Crude fiber
CE	Crude energy
Ca	Calcium
Fe	Iron
Mg	Magnesium
Na	Sodium
Zn	Zinc
EAA	Essential amino acids
Ala	Alanine
Glu	Glutamic acid
Leu	Leucine
Val	Valine
Arg	Arginine
Hist	Histidine
Lys	Lysine
Meth	Methionine
Isoleu	Isoleucine
Phy	Phenylanine
Thar	Threonine
DM	Dry matter
NDF	Neutral Detergent Fiber
ADF	Acid detergent fiber
AG	Fatty acids
NH3	Ammonia
HFL	House fly
Phy	Phosphorus
OM	Organic matter
HF	*Hermetia illucens*
AA	Amino acids
